# Cohort study investigating the effects of first stage of the English tobacco point-of-sale display ban on awareness, susceptibility and smoking uptake among adolescents

**DOI:** 10.1136/bmjopen-2016-012451

**Published:** 2017-01-23

**Authors:** Ilze Bogdanovica, Ann McNeill, John Britton

**Affiliations:** 1 Division of Epidemiology and Public Health, UK Centre for Tobacco and Alcohol Studies, University of Nottingham, Nottingham, UK; 2 UK Centre for Tobacco and Alcohol Studies, Addictions Department, Institute of Psychiatry, King's College London, London, UK

**Keywords:** smoking, point-of-sale displays, susceptibility to smoking

## Abstract

**Objective:**

A prospective evaluation of the effect of 2012 point-of-sale (PoS) display ban in supermarkets in England on perceived exposure to PoS displays, and on changes in susceptibility and smoking uptake among young people.

**Design:**

Cohort study.

**Settings:**

Seven schools in Nottinghamshire, England.

**Participants:**

1035 11–16-year-old school children.

**Primary and secondary outcome measures:**

Changes in reported exposure to PoS displays before and after prohibition, and the association between exposure to and awareness of PoS displays and change in susceptibility to smoking and smoking status between 2011 and 2012 (before the ban) and 2012 and 2013 (after the ban).

**Results:**

The proportion of children noticing tobacco PoS displays in supermarkets most or every time they visited a shop changed little between 2011 and 2012 (59.6% (95% CI 56.6% to 62.6%) and 58.8% (95% CI 55.8% to 61.8%), respectively); but decreased by about 13 percentage points to 45.7% (95% CI 42.7% to 48.7%) in 2013, after the ban. However, after adjusting for confounders, implementation of the first stage of the PoS ban in 2012 did not result in significant changes in the relation between susceptibility to smoking and smoking status and exposure to and awareness of PoS displays.

**Conclusions:**

Prohibition of PoS in large supermarkets resulted in a decline in the proportion of young people noticing PoS displays in large shops, but little or no change in smoking uptake or susceptibility. It remains to be seen whether extension of the PoS ban to all shops in 2015 has a more marked effect.

**Strengths and limitations of this study**

- This is the first individually linked cohort study to investigate changes in exposure to and awareness of tobacco point-of-sale (PoS) displays, and changes in susceptibility to smoking and smoking uptake in relation to first stage of tobacco PoS display ban in England.
- Data were collected using self-administered questionnaires including a wide range of variables: socio-demographic factors, smoking among peers and family, self-perceived academic performance and rebelliousness, smoking status and susceptibility to smoking, exposure to and awareness of tobacco PoS displays, and number of tobacco brands recognised.
- Our findings are limited by low power arising from the relatively small number of participants for whom linked data could be identified.
- Changes in susceptibility to smoking and smoking uptake were investigated 1 year after the implementation of the ban though longer follow-up time might be required to observe considerable changes in susceptibility as a result of reduced exposure and awareness.

## Introduction

Smoking is the largest avoidable cause of death in the UK.^1^ Although the prevalence of smoking among adults in Great Britain has declined substantially over recent decades,^2^ there are still about 9 million smokers in the UK,^3^ most of whom became smokers before the age of 18.^4^ Although smoking prevalence among young people in Britain has also declined, reaching 8% among 15-year olds in England in 2014, around 207 000 children start smoking every year in the UK.^4^ Therefore, policies to prevent smoking uptake among young people are of crucial public health importance.

Smoking prevalence has declined in the UK as a result of comprehensive tobacco control policies including legislation prohibiting most forms of tobacco advertising.^5^ However, until recently in the UK, this legislation provided an exemption for tobacco product displays at the point of sale (PoS). Previous studies have suggested that being exposed to tobacco PoS displays causes adults who intend to quit to make unplanned tobacco purchases,^6^ and that removal of PoS displays reduces these impulse purchases.^7^ Findings from the International Tobacco Control Four Country Survey support these findings, suggesting that PoS display bans reduce exposure to tobacco marketing and the frequency of unplanned purchases of tobacco products.^8^ Although there is less evidence on the effect of PoS displays on youth smoking behaviour, we have recently reported data from England suggesting that children with higher levels of exposure to tobacco PoS displays are more likely to be susceptible to smoking,^9^ and that noticing PoS displays more often was a prospective determinant of the onset of susceptibility (absence of a decision not to smoke).^10^ Being susceptible to smoking is associated with an increased risk of experimentation with smoking, and smoking uptake, among adolescents.^11^

In England, in April 2012, tobacco PoS displays were banned in all large shops, defined as those with a floor area over 280 square metres.^12^ We now report an extension to our earlier work^9^ ^10^ investigating whether this policy has reduced exposure to and awareness of tobacco at PoS among young people, or altered the previously observed relation between exposure to PoS displays and becoming susceptible to smoking or smoking uptake.

## Methods

### Data collection

Between March and May 2013, we carried out the third in a series of cross-sectional surveys (previously carried out in March–May 2011 and March 2012) of smoking behaviour, exposure to and awareness of PoS displays in students in years 7–11 in Nottinghamshire secondary schools.^9^ ^10^ Informed consent for school participation was obtained from head teachers, and opt-out consent for students by distributing forms to parents of all children in school years 7–11 (aged 11–16). All students whose parents and who themselves did not decline participation were invited to fill in a paper-based questionnaire under teacher supervision. Of the 11 schools surveyed in 2011, eight agreed to participate in 2012, and seven of these (and one other school which did not participate in 2012) provided data in 2013. As for this study, we linked data for students in 2011, 2012 and 2013, we were able to link data for all years for these seven schools. Further details on data collection are available elsewhere.^9^ ^10^

### Variables included

Our questionnaire collected information on demographic variables (age, sex and ethnicity); postcode, which was used to calculate Index for Multiple Deprivation quintiles as a measure of socioeconomic status; rebelliousness, self-perceived academic performance, smoking among family members and friends and whether smoking was allowed in the student's home. As in previous analyses of data from these surveys, 9 10 our main exposure variables were frequency of visiting shops; frequency of noticing PoS displays in these shops and the number of tobacco brands recognised. Questions about noticing PoS displays and visiting shops were asked separately for small shops and large shops and we looked at the changes in the proportion of children noticing PoS and visiting each type of shops between 2011 and 2012, and 2012 and 2013. Frequency of visiting shops was coded as a binary variable with two categories: at least two or three times a week and less than two or three times a week. Frequency of noticing also was coded into binary categories: sometime or less and most or every time. Number of brands recognised was coded into three distinct categories: none, 1–5 brands and more than 5 brands. Our main outcome variables were reported changes in susceptibility to smoking defined using previously validated questions by Pierce *et al*,^11^ ^13^ and change in smoking status from never-smoker to ever-smoker. Further details on the variables included are available in the paper reporting data from the 2011 and 2012 surveys.^10^ In this study, we investigated changes in children who provided data in all three surveys, and compared changes observed between 2011 and 2012, and between 2012 and 2013, to explore the effects of implementation of the PoS display ban in large shops.

### Analysis

We linked data on individual student responses in 2011, 2012 and 2013 using the student's name, school and school year. We explored changes in outcomes between 2011 and 2012 in relation to exposure variables and confounders in 2011 which captures pre-ban data, and then repeated the analysis looking at the changes in susceptibility and smoking status between 2012 and 2013 in relation to exposures in 2012 capturing changes following PoS display ban in large shops. We investigated these changes for small shops and large shops separately. We first investigated whether frequency of noticing PoS displays changed between three study years, and whether these changes differed between small and large shops. We then investigated whether changes in susceptibility to smoking and smoking uptake between 2011 and 2012 were related to exposure to and awareness of tobacco PoS displays in 2011, and whether changes between 2012 and 2013 were related to exposure to and awareness of tobacco PoS displays in 2012 after adjusting for potential confounders. We then compared these results to investigate whether association before and after implementation of PoS display ban in large shops and small shops differed. We used four main outcome variables: (1) the proportion of children who were non-susceptible never-smokers in 2011 and 2012 and became susceptible in the following year; (2) the proportion of children who were non-susceptible never-smokers in 2011 and 2012 and became smokers in the following year; (3) the proportion of children who were susceptible never-smokers and became smokers in subsequent year and (4) the proportion of children who were susceptible never-smokers and reverted to being non-susceptible never-smokers between 2011 and 2012, and 2012 and 2013. Students with missing values for outcome variables were excluded from the analysis; missing values for the exposure variables were included in the analysis as a separate category to maximise study power.

As in our previous analyses, we used multinomial logistic regression to estimate relative risk ratios for change in susceptibility and smoking status in relation to frequency of visiting shops, frequency of noticing PoS displays, number of brands recognised and variables that combine these. To allow for multiple testing, we set our statistical significance threshold at a probability of 1%, and calculated 99% CIs. We used a cluster sandwich estimator to account for clustering within classes and schools. Data were analysed using Stata V.11 (Stata Corp. College Station, Texas, USA).

## Results

From the seven schools participating in the 2011, 2012 and 2013 surveys, we received completed questionnaires from 4019, 3989 and 4014 participants, respectively. After excluding children who did not participate in all 3 years, and those with missing information on outcome variables, a cohort of 1035 children remained for analysis.

Overall, the proportion of children who were non-susceptible never-smokers decreased from year to year, from 77.5% in 2011, to 62.8% in 2012 and 55.3% in 2013. On the other hand, the proportion of children who were ever-smokers increased considerably from 4.5% in 2011 to 20.7% in 2013. 19.8% of children who were non-susceptible to smoking in 2011 became susceptible in 2012 and 6.0% became ever-smokers. Similar transitions were observed between 2012 and 2013 when 18.0% of those non-susceptible to smoking in 2012 became susceptible to smoking in 2013 and 5.2% became ever-smokers.

Table 1 displays summary data on a range of smoking and related variables from all 3 years and demonstrates little change in (for example) deprivation score, parental and sibling smoking, smoking in the family home, academic performance and rebelliousness; but identifies an increase in the number of friends who smoke, consistent with the overall increase in prevalence of ever-smoking within the cohort.

**Table 1:** Summary of 2011, 2012 and 2013 data for the 1035 participants with linked responses

Variable	2011 (n, %)	2012 (n, %)	2013 (n, %)
Sex			
Boy	503 (48.6)	503 (48.6)	503 (48.6)
Girl	532 (51.4)	532 (51.4)	532 (51.4)
Age			
11	147 (14.2)		
12	416 (40.2)	147 (14.2)	
13	379 (36.6)	434 (41.9)	105 (10.1)
14	90 (8.7)	365 (35.3)	406 (39.2)
15		88 (8.5)	420 (40.6)
16			101 (9.8)
Missing	3 (0.3)	1 (0.1)	3 (0.3)
Deprivation quintile			
1 (least deprived)	314 (30.3)	338 (32.7)	287 (27.6)
2	107 (10.3)	115 (11.1)	101 (9.7)
3	180 (17.4)	201 (19.4)	165 (15.9)
4	157 (15.2)	181 (17.5)	160 (15.4)
5 (most deprived)	132 (12.8)	151 (14.6)	130 (12.5)
Missing	145 (14.0)	49 (4.7)	197 (18.9)
Parental smoking			
Neither parent smokes	743 (71.8)	749 (72.4)	758 (73.2)
One parent smokes	192 (18.6)	188 (18.2)	202 (19.5)
Both parents smoke	90 (8.7)	76 (7.3)	71 (6.9)
Missing	10 (1.0)	22 (2.1)	4 (0.4)
Sibling smoking			
None smokes	963 (92.6)	928 (89.2)	934 (89.8)
At least one smokes	67 (6.4)	90 (8.7)	101 (9.7)
Missing	10 (1.0)	22 (2.1)	5 (0.5)
Smoking in the main family home			
Not allowed	870 (84.1)	893 (86.3)	896 (86.6)
Allowed	155 (15.0)	121 (11.7)	134 (13.0)
Missing	10 (1.0)	21 (2.0)	5 (0.5)
Number of smoking friends			
None	618 (59.7)	368 (35.6)	306 (29.6)
One or two	115 (11.1)	153 (14.8)	181 (17.5)
Three or more	94 (9.1)	233 (22.5)	290 (28.2)
Not sure	196 (19.0)	258 (24.9)	250 (24.2)
Missing	12 (1.2)	23 (2.2)	8 (0.8)
Self-perceived academic performance			
Excellent or good	830 (80.2)	794 (76.7)	781 (75.5)
Average or below average	185 (17.9)	220 (21.3)	249 (24.1)
Missing	20 (1.9)	21 (2.0)	5 (0.5)
Rebelliousness			
Low	592 (57.2)	593 (57.3)	619 (59.8)
High	420 (40.6)	395 (38.2)	390 (37.7)
Missing	23 (2.2)	47 (4.5)	26 (2.5)
Susceptibility to smoking			
Non-susceptible never-smoker	802 (77.5)	650 (62.8)	572 (55.3)
Susceptible never-smoker	186 (18.0)	250 (24.2)	249 (24.1)
Ever-smoker	47 (4.5)	135 (13.0)	214 (20.7)
Notice cigarettes on displays in large shops			
Sometimes or less	401 (38.7)	388 (37.5)	524 (50.6)
Most times or every time	617 (59.6)	609 (58.8)	473 (45.7)
Missing	17 (1.6)	38 (3.7)	38 (3.7)
Notice cigarettes on displays in small shops			
Sometimes or less	259 (25.0)	210 (20.3)	290 (28.0)
Most times or every time	745 (72.0)	774 (74.8)	697 (67.3)
Missing	31 (3.0)	51 (4.9)	48 (4.6)
Frequency of visiting large shops			
Less than 2 or 3 times a week	360 (34.8)	356 (34.4)	344 (33.2)
At least 2 or 3 times a week	667 (64.4)	669 (64.6)	689 (66.6)
Missing	8 (0.8)	10 (1.0)	2 (0.2)
Frequency of visiting small shops			
Less than 2 or 3 times a week	485 (46.9)	493 (47.6)	435 (42.0)
At least 2 or 3 times a week	540 (52.2)	535 (51.7)	598 (57.8)
Missing	10 (1.0)	7 (0.7)	2 (0.2)
Number of brands recognised			
None	282 (27.3)	232 (22.4)	227 (21.9)
1–5 brands	381 (36.8)	362 (35.0)	365 (35.3)
More than 5 brands	239 (23.1)	346 (33.4)	356 (34.4)
Missing	133 (12.9)	95 (9.2)	87 (8.4)

The proportion of children who reported noticing tobacco PoS displays most or every time they visited a supermarket remained stable in 2011 and 2012 (59.6% and 58.8%, respectively) but fell slightly to 45.7% in 2013 after implementation of the PoS display ban in large shops. There was also a small reduction in the proportion of children noticing PoS displays most or every time they visited a small shop from 74.8% in 2012 to 67.3%% in 2013 though the frequency of visiting shops remained stable (see table 1).

### Changes in smoking susceptibility and status in relation to exposure variables at univariable level

Analysis at univariable level suggested that among those who were non-susceptible to smoking in 2011 the risk of becoming susceptible to smoking in 2012 was higher among students with lower levels of self-perceived academic performance, higher levels of rebelliousness and visited large shops less frequently, but recognised a higher number of brands (table 2). The risk of becoming susceptible to smoking in 2013 among non-susceptible never-smokers in 2012 was higher among students who visited large shops less frequently, among those living in homes where smoking was allowed and those who recognised a higher number of tobacco brands (table 3).

**Table 2:** Unadjusted relative risk ratios for changes in susceptibility and smoking status in relation to explanatory variables 2011–2012

	Among non-susceptible never-smokers at baseline						Among susceptible never-smokers at baseline					
	RRR of becoming susceptible			RRR of becoming an ever-smoker			RRR of becoming non-susceptible			RRR of becoming an ever-smoker		
	Estimate	99% CI	p Value	Estimate	99% CI	p Value	Estimate	99% CI	p Value	Estimate	99% CI	p Value
Age												
11	1.00			1.00			1.00			1.00		
12	0.90	0.40 to 2.03	0.746	2.62	0.55 to 12.51	0.114	0.87	0.17 to 4.53	0.829	0.46	0.14 to 1.57	0.105
13	0.97	0.49 to 1.91	0.894	5.94	1.33 to 26.49	0.002	0.31	0.07 to 1.36	0.041	0.74	0.36 to 1.50	0.271
14	1.03	0.51 to 2.10	0.908	9.29	1.49 to 57.78	0.002	0.62	0.27 to 1.43	0.137	0.98	0.37 to 2.62	0.968
15												
Parental smoking												
Neither parent smokes	1.00			1.00			1.00			1.00		
One parent smokes	1.73	0.99 to 3.04	0.011	2.94	0.83 to 10.43	0.028	1.84	0.61 to 5.59	0.158	2.68	1.27 to 5.64	0.001
Both parents smoke	1.37	0.48 to 3.96	0.441	3.29	1.06 to 10.24	0.007	1.17	0.22 to 6.27	0.814	2.61	1.78 to 5.77	0.002
Sibling smoking												
None smokes	1.00			1.00			1.00			1.00		
At least one smokes	1.57	0.51 to 4.86	0.303	2.42	0.62 to 9.53	0.096	1.38	0.33 to 5.80	0.568	3.77	1.10 to 12.84	0.005
Smoking in the main family home												
Not allowed	1.00			1.00			1.00			1.00		
Allowed	1.63	0.81 to 3.27	0.073	4.51	2.69 to 7.56	<0.001	1.23	0.33 to 4.56	0.683	1.20	0.31 to 4.72	0.727
Number of friends who smoke												
None	1.00			1.00			1.00			1.00		
One or two	1.97	0.80 to 4.87	0.055	2.85	0.58 to 14.07	0.090	0.58	0.10 to 3.44	0.415	1.03	0.24 to 4.39	0.961
Three or more	2.32	0.70 to 7.68	0.069	8.23	4.32 to 15.65	<0.001	0.41	0.05 to 3.36	0.276	2.67	1.06 to 6.73	0.006
Not sure	1.66	1.07 to 2.60	0.003	3.12	0.94 to 10.32	0.014	0.62	0.24 to 1.61	0.129	1.16	0.52 to 2.58	0.641
Self to perceived academic performance												
Excellent or good	1.00			1.00			1.00			1.00		
Average or below average	2.03	1.04 to 3.94	0.006	1.90	0.92 to 3.92	0.023	0.53	0.21 to 1.31	0.070	0.81	0.42 to 1.56	0.413
Rebelliousness												
Low	1.00			1.00			1.00			1.00		
High	1.73	1.27 to 2.35	<0.001	3.78	2.78 to 5.14	<0.001	0.87	0.55 to 1.39	0.452	2.17	0.63 to 7.51	0.107
Noticing point-of-sale displays in large shops												
Sometimes or less	1.00			1.00			1.00			1.00		
Most or every time	1.37	0.95 to 1.96	0.027	3.17	0.92 to 10.93	0.017	0.97	0.50 to 1.90	0.911	0.95	0.49 to 1.85	0.850
Frequency of visiting large shops												
Less than 2 or 3 times a week	1.00			1.00			1.00			1.00		
At least 2 or 3 times a week	0.58	0.43 to 0.78	<0.001	0.63	0.31 to 1.25	0.080	1.03	0.57 to 1.86	0.906	0.73	0.40 to 1.35	0.193
Noticing point-of-sale displays in small shops												
Sometimes or less	1.00			1.00			1.00			1.00		
Most or every time	1.23	0.93 to 1.63	0.062	2.42	1.17 to 5.00	0.002	0.66	0.20 to 2.22	0.377	1.39	0.63 to 3.08	0.279
Frequency of visiting small shops												
Less than 2 or 3 times a week	1.00			1.00			1.00			1.00		
At least 2 or 3 times a week	0.93	0.73 to 1.18	0.433	0.36	0.29 to 0.46	<0.001	1.01	0.56 to 1.84	0.950	0.37	0.12 to 1.11	0.020
Number of brands recognised												
None	1.00			1.00			1.00			1.00		
1–5	1.64	0.87 to 3.08	0.044	2.24	0.91 to 5.53	0.021	0.98	0.25 to 3.82	0.976	2.05	0.71 to 5.94	0.081
More than 5	2.67	1.65 to 4.33	<0.001	6.07	2.38 to 15.46	<0.001	0.60	0.20 to 1.78	0.227	2.78	0.75 to 10.32	0.044

Sex and quintiles of Index of Multiple Deprivation not presented as these were not significant predictors for any of the outcome variables.

RRR, relative risk ratios.

**Table 3:** Unadjusted relative risk ratios for changes in susceptibility and smoking status in relation to explanatory variables 2012–2013

	Among non-susceptible never-smokers at baseline						Among susceptible never-smokers at baseline					
	RRR of becoming susceptible			RRR of becoming an ever-smoker			RRR of becoming non-susceptible			RRR of becoming an ever-smoker		
	Estimate	99% CI	p Value	Estimate	99% CI	p Value	Estimate	99% CI	p Value	Estimate	99% CI	p Value
Smoking in the main family home												
Not allowed	1.00			1.00			1.00			1.00		
Allowed	2.00	1.11 to 3.60	0.002	4.11	1.05 to 16.14	0.008	1.18	0.20 to 6.84	0.813	1.85	0.68 to 4.99	0.111
Number of friends who smoke												
None	1.00			1.00			1.00			1.00		
One or two	1.51	0.73 to 3.11	0.144	1.94	0.89 to 4.26	0.029	0.75	0.17 to 3.30	0.623	2.48	0.36 to 17.08	0.225
Three or more	1.67	0.65 to 4.26	0.161	6.38	1.63 to 25.04	<0.001	0.57	0.15 to 2.15	0.278	3.09	0.43 to 22.11	0.140
Not sure	1.14	0.60 to 2.14	0.604	2.55	1.05 to 6.21	0.007	0.57	0.24 to 1.35	0.090	2.43	0.37 to 16.01	0.226
Self-perceived academic performance												
Excellent or good	1.00			1.00			1.00			1.00		
Average or below average	1.03	0.48 to 2.22	0.923	2.11	1.03 to 4.33	0.007	1.40	0.87 to 2.27	0.070	1.40	0.68 to 2.89	0.230
Rebelliousness												
Low	1.00			1.00			1.00			1.00		
High	1.89	0.77 to 4.65	0.068	2.82	0.99 to 7.99	0.011	1.09	0.59 to 2.04	0.710	1.48	0.64 to 3.47	0.230
Noticing point-of-sale displays in large shops												
Sometimes or less	1.00			1.00p			1.00			1.00		
Most or every time	0.79	0.52 to 1.18	0.128	1.01	0.43 to 2.38	0.966	0.52	0.33 to 0.81	<0.001	1.85	0.72 to 4.72	0.093
Frequency of visiting large shops												
Less than 2 or 3 times a week	1.00			1.00			1.00			1.00		
At least 2 or 3 times a week	0.56	0.33 to 0.96	0.005	0.41	0.17 to 0.97	0.008	0.74	0.39 to 1.40	0.226	0.54	0.32 to 0.93	0.003
Noticing point-of-sale displays in small shops												
Sometimes or less	1.00			1.00			1.00			1.00		
Most or every time	0.80	0.42 to 1.54	0.383	0.89	0.36 to 2.20	0.736	0.79	0.25 to 2.52	0.603	1.38	0.37 to 5.12	0.532
Frequency of visiting small shops												
Less than 2 or 3 times a week	1.00			1.00			1.00			1.00		
At least 2 or 3 times a week	1.01	0.61 to 1.68	0.952	0.36	0.11 to 1.17	0.025	0.90	0.27 to 3.03	0.829	0.23	0.09 to 0.55	<0.001
Number of brands recognised												
None	1.00			1.00			1.00			1.00		
1–5	1.20	0.69 to 2.09	0.406	1.14	0.34 to 3.79	0.781	0.40	0.15 to 1.07	0.016	1.07	0.38 to 3.04	0.859
More than 5	1.81	1.29 to 2.54	<0.001	2.02	0.50 to 8.14	0.195	0.53	0.12 to 2.25	0.256	1.83	0.59 to 5.69	0.170

Sex, age, quintiles of Index of Multiple Deprivation, parental smoking and sibling smoking not presented as these were not significant predictors for any of the outcome variables.

RRR, relative risk ratios.

An increased risk of becoming an ever-smoker in 2012 among those who were non-susceptible never-smokers in 2011 was associated with age, both parents being smokers, greater number of smoking friends, higher levels of rebelliousness, noticing PoS displays in small shops more often, recognising greater number of tobacco brands and lower frequency of visiting small shops. Among those who were non-susceptible to smoking in 2012, the risk of becoming an ever-smoker in 2013 was higher among children with a greater number of smoking friends, for whom smoking was allowed in their main home, those with lower levels of self-perceived academic performance and those who visited large shops less frequently.

Among children who were susceptible to smoking in 2011, the risk of becoming an ever-smoker was higher among children with smoking parents and siblings and greater number of smoking friends, though among those who were susceptible to smoking in 2012 the risk of becoming an ever-smoker in 2013 was associated with having smoking siblings and visiting large and small shops less frequently (table 3).

### Changes in smoking susceptibility and status in relation to exposure variables at multivariable level

In a multivariable analysis with adjustment for confounding by age, sex, deprivation, parental smoking, sibling smoking, smoking in the main family home, number of smoking friends, self-perceived academic performance and rebelliousness, the risk of becoming susceptible to smoking in 2012 among those who were non-susceptible never-smokers in 2011 was unrelated to main exposure variables (frequency of visiting small and large shops and frequency of noticing PoS displays in large and small shops) though recognising five or more tobacco brands was associated with a twofold risk of becoming susceptible in 2012 (table 4). However, none of the exposure variables were related to becoming susceptible in 2013 among children who were non-susceptible to smoking in 2012. Similarly, exposure variables other than recognising more than five tobacco brands in 2011 were unrelated to becoming a smoker among children who were non-susceptible never-smokers in 2011 and 2012. Also, none of the main exposure variables were related to becoming a smoker either in 2012 or 2013 among children who were susceptible to smoking in 2011 and 2012 (table 5).

**Table 4:** Adjusted relative risk ratios for changes in susceptibility in relation to noticing point-of-sale displays, frequency of visiting shops and number of brands recognised between 2011–2012 and 2012–2013

	Among non-susceptible never-smokers at baseline 2011–2012						Among non-susceptible never-smokers at baseline 2012–2013					
	RRR of becoming susceptible			RRR of becoming an ever-smoker			RRR of becoming susceptible			RRR of becoming an ever-smoker		
	Estimate	99% CI	p Value	Estimate	99% CI	p Value	Estimate	99% CI	p Value	Estimate	99% CI	p Value
Noticing point-of-sale displays in large shops												
Sometimes or less	1.00			1.00			1.00			1.00		
Most or every time	1.31	0.81 to 2.12	0.153	2.72	1.00 to 7.40	0.010	0.79	0.45 to 1.36	0.254	0.98	0.38 to 2.50	0.954
Frequency of visiting large shops												
Less than 2 or 3 times a week	1.00			1.00			1.00			1.00		
At least 2 or 3 times a week	0.62	0.38 to 1.01	0.011	0.76	0.33 to 1.75	0.394	0.61	0.34 to 1.07	0.023	0.48	0.18 to 1.22	0.043
Noticing point-of-sale displays in small shops												
Sometimes or less	1.00			1.00			1.00			1.00		
Most or every time	1.19	0.70 to 2.03	0.391	2.27	0.76 to 6.85	0.055	0.76	0.40 to 1.44	0.269	0.81	0.27 to 2.43	0.618
Frequency of visiting small shops												
Less than 2 or 3 times a week	1.00			1.00			1.00			1.00		
At least 2 or 3 times a week	1.00	0.62 to 1.61	0.994	0.44	0.19 to 1.02	0.012	1.12	0.64 to 1.95	0.601	0.42	0.16 to 1.12	0.023
Number of brands recognised												
None	1.00			1.00			1.00			1.00		
1–5	1.61	0.85 to 3.02	0.054	2.11	0.64 to 6.96	0.106	1.18	0.58 to 2.40	0.555	1.06	0.31 to 3.60	0.908
More than 5	2.49	1.23 to 5.02	0.001	4.96	1.51 to 16.34	0.001	1.60	0.75 to 3.44	0.110	1.47	0.41 to 5.29	0.437

RRR, relative risk ratios.

**Table 5:** Adjusted relative risk ratios for changes in susceptibility in relation to noticing point-of-sale displays, frequency of visiting shops and number of brands recognised between 2011–2012 and 2012–2013

	Among susceptible never-smokers at baseline 2011–2012						Among susceptible never-smokers at baseline 2012–2013					
	RRR of becoming non-susceptible			RRR of becoming an ever-smoker			RRR of becoming non-susceptible			RRR of becoming an ever-smoker		
	Estimate	99% CI	p Value	Estimate	99% CI	p Value	Estimate	99% CI	p Value	Estimate	99% CI	p Value
Noticing point-of-sale displays in large shops												
Sometimes or less	1.00			1.00			1.00			1.00		
Most or every time	0.84	0.32 to 2.18	0.635	0.97	0.34 to 2.72	0.935	0.52*	0.22 to 1.23	0.05	1.85*	0.63 to 5.45	0.144
Frequency of visiting large shops												
Less than 2 or 3 times a week	1.00			1.00			1.00			1.00		
At least 2 or 3 times a week	1.04	0.41 to 2.68	0.910	0.75	0.27 to 2.06	0.468	0.74*	0.33 to 1.68	0.344	0.54*	0.24 to 1.24	0.056
Noticing point-of-sale displays in small shops												
Sometimes or less	1.00			1.00			1.00			1.00		
Most or every time	0.51	0.17 to 1.53	0.113	1.34	0.34 to 5.27	0.557	0.52*	0.22 to 1.23	0.050	1.85*	0.63 to 5.45	0.144
Frequency of visiting small shops												
Less than 2 or 3 times a week	1.00			1.00			1.00			1.00		
At least 2 or 3 times a week	1.14	0.46 to 2.82	0.716	0.35	0.12 to 1.02	0.012	0.79*	80.30 to 2.08	0.532	1.38*	0.46 to 4,15	0.457
Number of brands recognised												
None	1.00			1.00			1.00			1.00		
1–5	0.98	0.29 to 3.26	0.958	2.00	0.39 to 10.15	0.272	0.40*	0.13 to 1.20	0.031	1.07*	0.28 to 4.09	0.890
More than 5	0.57	0.15 to 2.23	0.293	2.66	0.52 to 13.60	0.123	0.53*	0.10 to 2.74	0.317	1.83*	0.51 to 6.62	0.227

*The final model was based on univariate relationship.

RRR, relative risk ratios.

## Discussion

To the best of our knowledge, this is the first individually linked cohort study to explore changes in susceptibility to smoking, and smoking status, in relation to the removal of tobacco PoS displays from supermarkets and other large retailers in the UK; and hence, the first to evaluate the associations between exposure and changes in susceptibility and smoking status before and after the introduction of the ban on PoS displays in supermarkets and other large shops. Our findings suggest that there was a reduction in the proportion of children noticing tobacco PoS displays after the ban was implemented. However, while our findings at univariable level suggest that children who noticed PoS displays more often were more likely to become susceptible to smoking and to become smokers, we did not find a statistically significant independent effect once potential confounders were taken into account. In this respect, there was no difference in the results we obtained before and after the ban, when associations between main exposures and outcomes were consistently non-significant.

Our study findings are limited by low power arising from the small number of participants for whom linked data from all three surveys were available, and the small number of individuals making the progression to smoking susceptibility or uptake. Also, the fact that the cohort ages over study period makes it difficult to assess whether smoking uptake rates have changes as a response to the implementation of the first stage of tobacco PoS display ban. Owing to the fact that some of the schools did not participate in one or more survey waves, we were able to link data from only seven out of the initial 11 schools, and linkage proved impossible for many participants as a result of missing or incomplete identity information. However, the demographic characteristics of the children we were able to link for all 3 years were broadly similar, particularly in relation to deprivation, to those of the full original sample of participants in the 2011 survey^9^ and of 2012 participants.^10^ Another important limitation is that we were asking children about their exposure to and awareness of PoS displays separately for small shops (corner shops/newsagents and off licences) and for supermarkets (large shops), but cannot be sure that respondents were able to differentiate these two types of shops. For example, Tesco is typically known as a supermarket in the UK and has local stores which were sufficiently small to be excluded from the 2012 PoS prohibition. Although we do not have information on compliance with tobacco PoS display ban in large shops in England, recent evidence from Scotland suggests that compliance with ban in small shops was high^14^ and we believe it would be generalisable to first stage of PoS display ban in English settings.

We measured changes in susceptibility and smoking status 1 year before and 1 year after the large retailer PoS display ban was implemented in England, and it is possible that a longer period may have had more substantial effects on children's smoking. We selected the measures that, to the best of our knowledge, were best likely to capture changes in exposure to and awareness of tobacco PoS displays, but it is possible that these measures were insufficiently sensitive to capture immediate effects of the PoS display ban. Although our findings relate to children's smoking, they are consistent with data from Ireland where there was no immediate decrease in general smoking prevalence after implementation of a PoS ban.^15^ However, the ban in Ireland led to a reduction in perceived smoking prevalence among young people and adults, suggesting that removal of PoS displays made not smoking easier.^15^

Cross-sectional and linked data from earlier waves of this cohort study clearly indicated that exposure to and awareness of tobacco PoS displays was associated with increased risk of becoming susceptible to smoking and also becoming a smoker.^9^ ^10^ Previous studies elsewhere have also consistently suggested that being exposed to tobacco PoS promotion leads to increased likelihood of becoming susceptible to smoking, experimenting with smoking or becoming regular or occasional smoker.^16^ ^17^ Although this tobacco policy is primarily aimed at reducing smoking uptake among children, it appears to have an effect on adult smoking by reducing the number of impulse purchases in jurisdictions where PoS bans are implemented.^18^ Evaluation of the Irish tobacco PoS display ban suggested that removal of PoS displays had a potential to de-normalise smoking and young people felt that it could make it easier for them to abstain from smoking uptake.^15^ Similarly, in Norway, a PoS display ban implemented in 2010 was perceived as a barrier limiting access to tobacco products affecting brand attachment and therefore leading to de-normalisation of smoking.^19^

Evidence from previous research suggests that the 2012 partial PoS display ban had no immediate effect on smoking prevalence and cigarette consumption among adults, though a steeper reduction in prevalence was observed over the 3 years following the ban.^17^ However, a recent study exploring the effects of PoS display bans in New Zealand suggests that implementation of the ban led to a reduction in initiation, experimentation and regular smoking among young people.^20^ Our findings indicate, however, that while prohibition of PoS tobacco displays in large shops in England reduced the proportion of young people reporting exposure to the displays in large and small shops, their removal did not result in a significant reduction in smoking behaviour among young people. Further work is required to determine whether removal of PoS displays in smaller shops, which tend to be the greater source of exposure of young people and which were afforded an exclusion from the English PoS prohibition until April 2015 has yielded a greater effect.
